# Simple Method to Improve Electrical Conductivity of Films Made from Single-Walled Carbon Nanotubes

**DOI:** 10.3390/nano9081113

**Published:** 2019-08-02

**Authors:** Bogumiła Kumanek, Tomasz Wasiak, Grzegorz Stando, Paweł Stando, Dariusz Łukowiec, Dawid Janas

**Affiliations:** 1 Department of Organic Chemistry, Bioorganic Chemistry and Biotechnology, Silesian University of Technology, B. Krzywoustego 4, 44-100 Gliwice, Poland; 2 Faculty of Mechanical Engineering, Silesian University of Technology, Konarskiego 18, 44-100 Gliwice, Poland

**Keywords:** carbon nanotubes, electrical conductivity, thermal conductivity

## Abstract

Despite the widespread use of sonication for individualization of nanomaterials, its destructive nature is rarely acknowledged. In this study, we demonstrated how exposure of the material to a hostile sound wave environment can be limited by the application of another preprocessing step. Single-walled carbon nanotubes (CNTs) were initially ground in a household coffee grinder, which enabled facile deagglomeration thereof. Such a simple approach enabled us to obtain high-quality CNT dispersion at reduced sonication time. Most importantly, electrical conductivity of free-standing films prepared from these dispersion was improved almost fourfold as compared with unground material eventually reaching 1067 ± 34 S/cm. This work presents a new approach as to how electrical properties of nanocarbon ensembles may be enhanced without the application of doping agents, the presence of which is often ephemeral.

## 1. Introduction

Carbon nanotubes (CNTs) have shown exceptional electrical [1,2], thermal [3,4,5], mechanical [6], and optical properties [7,8], and so the interest in these materials has been growing year by year. Particular attention has been devoted to enhance their electrical characteristics because of their unique potential to replace copper and aluminum in electrical wiring [9]. Another important aspect is that conductive CNT ensembles can be used for electrical stimulation in medicine [10,11]. However, to reach the technology readiness level appropriate for their deployment in real-life, electrical conductivity of these materials still needs to be improved. That is a multidimensional problem, which results from a range of technological constraints, which need to be tackled. Firstly, electrical conductivity of individual CNTs depends on the way they are “rolled-up” from a graphene sheet [12], so appropriate methods of structure control have to be established (either at the synthesis stage or during post-processing [13]). Secondly, CNTs should be assembled into networks such as fibers, wires or coatings in the right way [9]. To obtain high-performance material, CNTs should ideally be composed of just metallic chiralities, which are long and aligned along the expected direction of current flow. Thirdly, since CNTs have a limited length, measures have to be taken to alleviate the problem of contact resistance. This is often done by doping with chemical compounds (chemically or physically) [14,15,16,17,18] or by decoration with metal nanoparticles [19].

The literature contains information that the electrical conductivity of aligned, condensed and/or doped CNT films may reach the level of 10,000 S/cm [20,21]. Despite their merits, it is still technologically challenging to produce such aligned CNT networks at appreciable scale. Most commonly, isotropic CNT films are produced and unfortunately they have inferior electrical conductivity. The presence of impurities or improper charge transfer often push their electrical conductivity below 500 S/cm [13,22,23]. That is why new solutions are being searched for in order to make full use of the potential existing in CNTs.

Free-standing CNT films may be obtained using one of the numerous methods available [24] such as dip-coating [25], spin-coating [26], spray-coating [27], membrane filtration [28], printing [29], and electrophoretic deposition [30]. What all these methods have in common is that they require proper dispersion of CNTs. To eliminate the problem of aggregates, CNTs either have to be modified chemically or a surfactant has to be introduced. Such mixture is then agitated in a selected solvent using sonication [31,32] or shear mixing [33]. Both methods have certain drawbacks. In the case of shear mixing, it is necessary to apply long mixing periods of as much as 20h [31] or increase the shearing rate to reduce the dispersion time, which may have a negative impact on the conductivity of the material [34]. In the case of sonication, it has been proven that it has a destructive influence on CNTs, which was first observed by Green et al. in 1996 and confirmed using TEM analysis [35]. Later on, subsequent publications emerged that confirmed the dark side of sonication [31,32,36,37,38], which is rarely acknowledged. There are two methods of sonication: tip and bath. Both have a negative effect on CNTs, but the destructive impact of tip sonication is greater, because sound energy is concentrated [32,39,40]. Arnold and co-workers [31] demonstrated that tip sonication time also affects the average length of CNTs. The increase in sonication time from 10 to 60 min resulted in a reduction in the length of CNTs by as much as 35%. Since the length of CNTs is reduced, an increase in the number of junctions between them is observed, which in the end decreases the overall electrical conductivity of the network [36]. Nevertheless, individualization of CNTs for certain applications is required. Unfortunately, van der Waals forces between individual CNTs (especially of single-walled nature) are relatively strong and commonly result in the formation of bundles. They are hard to dismantle without resorting to the aforementioned high power methods.

In this work, we developed a method of pre-processing of single-walled CNT material to enhance the electrical conductivity of the films made from them by almost four times (eventually reaching the electrical conductivity of 1067 ± 34 S/cm). To reduce the duration of sonication, which would otherwise have a negative impact on the properties of the material, we first deagglomerated the parent CNT powder by using a household coffee grinder (with a price tag of about 20 EUR). This enabled us to obtain a proper dispersion of the material in a short time, which resulted in a significant increase in electrical conductivity. In order to arrive at the complete picture of the impact of parameters on the properties of obtained CNT films, in our research we used three different periods of grinding (5, 15 and 60 min). Moreover, we also explored the influence of the content of ethyl cellulose (its ratio to CNTs amounted to 1:1, 1:10 and 1:50 by weight, respectively). We found out that although it is removed by thermal annealing, its content exerts an effect on the properties of the final material.

## 2. Materials and Methods

Free-standing films were obtained from high quality single walled CNTs (Tuball^TM^, Leudelange, Luxembourg) by a method previously reported by us [41]. Briefly, the method consisted of adding a suitable amount of ethyl cellulose (EC, ethoxyl content 48%, 22 cps Acros Organics, Geel, Belgium) to a mixture containing equal amounts of ice cold acetone (pure p.a. VWR Chemicals, Radnor, PA, USA) and toluene (pure p.a. Chempur, Piekary Slaskie, Poland); 0.25 g, 0.025g and 0.005 g were introduced for films of type 1:1, 1:10 and 1:50, respectively. After complete dissolution of EC, 0.25 g of CNT was added to the mixture. Then, the mixture was sonicated using UP200ST sonicator (Hielscher, Teltow, Germany) with an amplitude of 100%, until arriving at a uniform mixture. The sonication time was ca. 5 min. The mixture was then placed onto a Kapton foil substrate, allowing the solvent to slowly evaporate and form the film. Lastly, the film was removed from the substrate and EC was removed by thermal annealing in air where indicated. Its rapid combustion, which lasts no more than a few of seconds, does not leave residue in the final material.

The following preliminary processing of CNTs was employed. CNTs (0.25 g) were ground in a 200W coffee grinder (Profi Cook PC-KSW 1021, Opole, Poland) presented in Figure S1. Three periods of grinding were applied (using proper grinding time intervals advised by the manufacturer): 5 min, 15 min and 60 min. As a reference, we have also used unground CNTs for the study.

**Figure 1:**
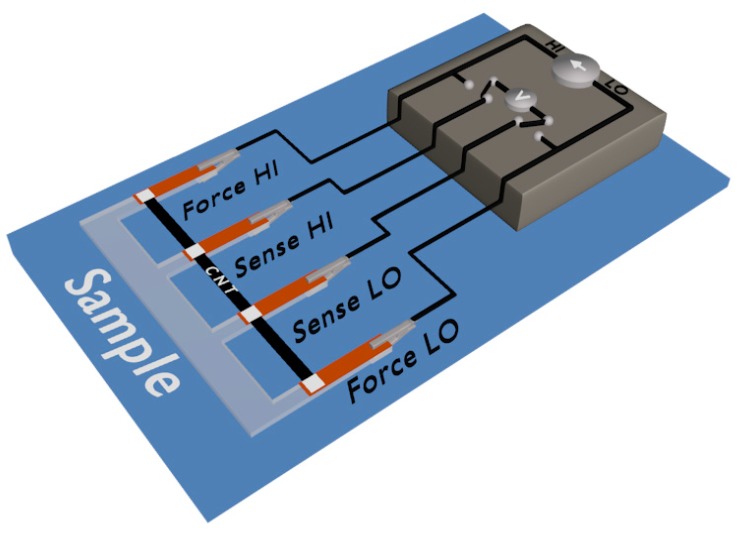
Scheme of setup for measurement of electrical and thermal conductivity of CNT films.

The obtained films were characterized using Raman spectroscopy (Renishaw, Wotton-under-Edge, UK) in order to determine the influence of grinding duration and the amount of EC on the structure of the films. A laser with a wavelength of λ = 514 nm was applied to record the spectra from 0 to 3500 cm^−1^. In order to reduce the background noise, accumulation time was 10 s. Moreover, 10 acquisitions were used for each sample (five different locations were analyzed in every case). Photographs were taken using a camera (Nikon DSLR D3200 with a AF-S DX NIKKOR 18-105 mm lens, Tokyo, Japan) in order to demonstrate the differences among the films seen with the naked eye. SEM micrographs were obtained using Scanning Electron Microscope (Supra 35, Carl Zeiss, Oberkochen, Germany) at 5 kV acceleration voltage. Due to the conductive nature of the CNT films, they were not sputtered with metal. Electrical conductivity was determined using a 4-probe method (Keithley 2450 SourceMeter, Cleveland, OH, USA). Thermal conductivity was obtained by means of a steady-state method with IR thermal camera (FLIR ETS 320, Wilsonville, OR, USA) to record the temperature gradient ca. 5 °C under bias voltage of 0.25 V. In order to measure both types of conductivity as absolute values, we prepared custom-made sample holders (Figure 1).

Four copper electrodes were situated at equal distances on an insulating substrate (glass slides). CNT films of the dimensions of ca. 3 mm × 60 mm × 0.2 mm were cut out from the film, which were then placed on the copper electrodes using silver paint to eliminate contact resistance. The film thickness was measured using an Electronic Universal micrometer LINEAR 0–0.25 mm/0.001 mm (Dunstable, UK). At least five measurements were made for each sample. Five samples were tested for each combination of parameters. The results were averaged and the statistical error was calculated (presented as standard deviation).

## 3. Results and Discussion

**Figure 2:**
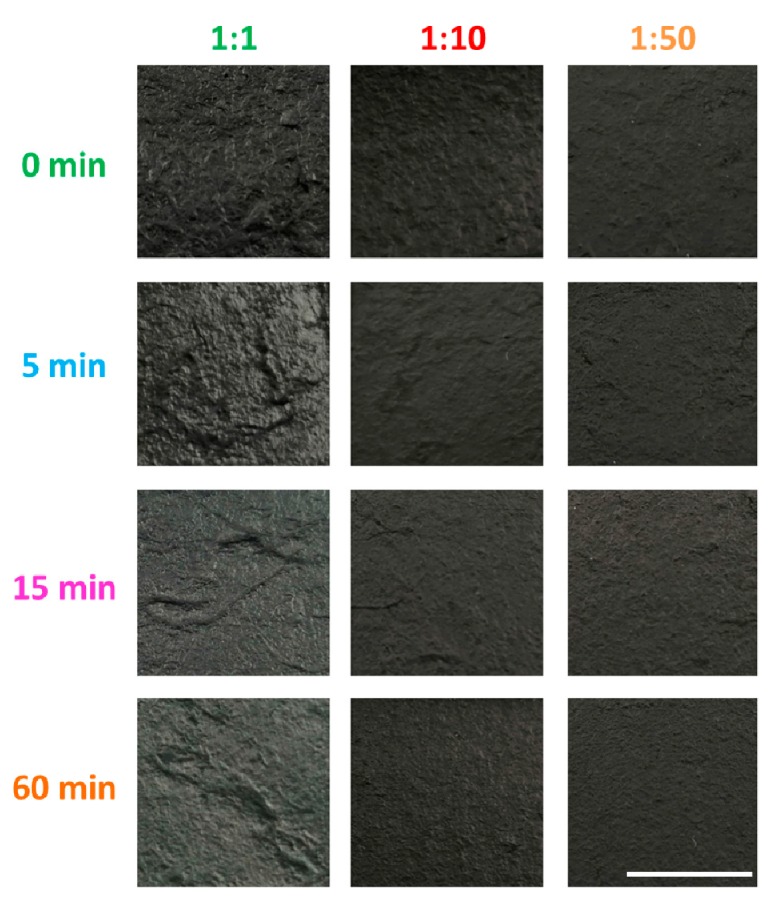
Photographs of all CNT films (with EC). All the photographs were taken at the same magnification and the white marker is 1 cm long.

We started the study by visual analysis of the surface of the films (Figure 2). Depending on the applied amount of EC and duration of grinding, differences can be observed with the naked eye. The films obtained from equal amounts of EC and CNTs (1:1) are characterized by high surface roughness. Moreover, some degree of CNT agglomeration can be noticed. To tackle this problem, we decided to lower the amount of EC down to 1:10 and 1:50 (relative to the content of CNTs). This approach had a positive effect on the smoothness of the films obtained (especially in the case of 1:50 CNT film). Additionally, it can be observed that grinding of CNTs has a favorable influence on the quality of the film. The longer the time of grinding, the more uniform the surface is. This is most obvious when the amount of EC is lowered to 1:10 or 1:50. In the case of 1:1, CNT agglomerates are evident regardless of the grinding time.

**Figure 3:**
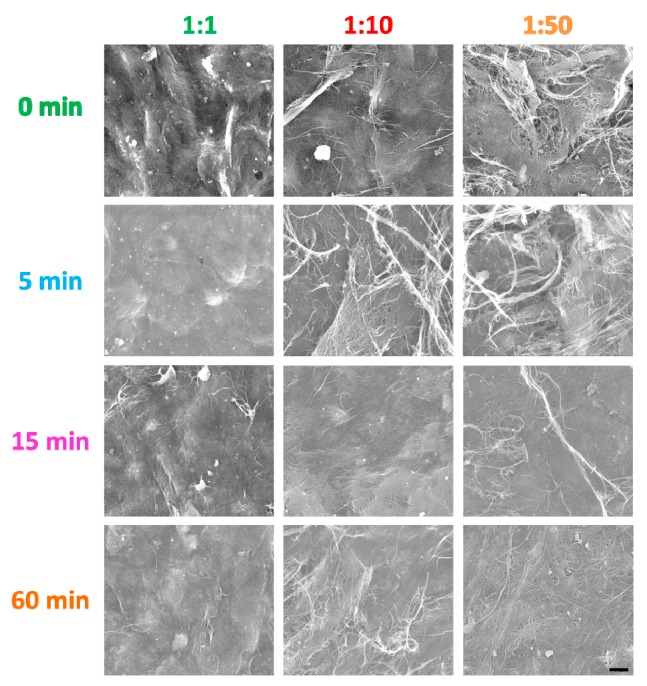
SEM micrographs for all the CNT-EC films made using the same magnification. The black marker is 1 µm long.

To get a more precise insight into the microstructure of the material, we obtained SEM micrographs for all the CNT film samples (Figure 3). It is evident that with a decrease of EC content from 1:1 to 1:50, the individual CNTs and their bundles appear more pronounced (for the unground samples or samples ground for 5 min). Prolonged grinding time (15 min and 60 min) decreased the amount of agglomerates for all the explored contents of EC to CNTs. On another note, protruding CNTs and their bundles can be observed in some of the images. This once again demonstrates complexity in the accurate determination of the thickness and density of CNT macrostructures [9,42].

**Figure 4:**
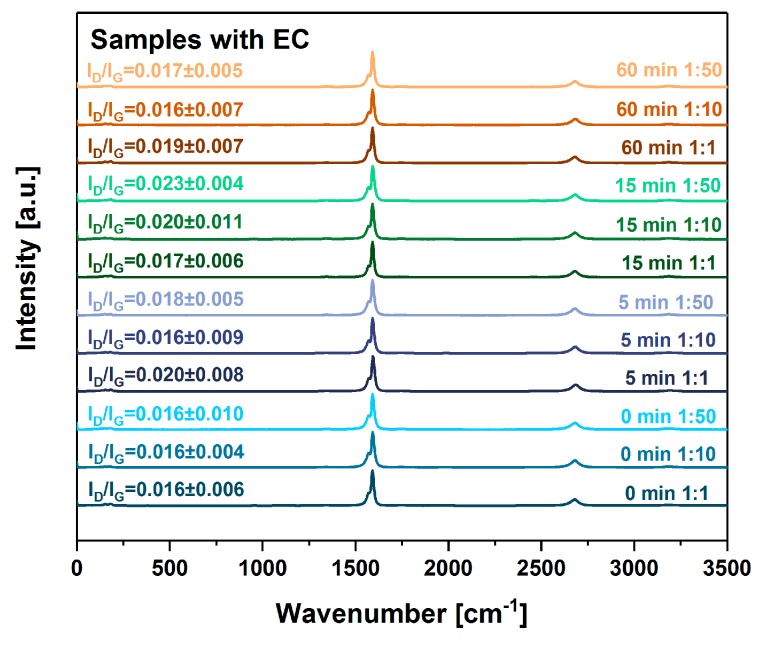
The influence of grinding time and EC content on crystallinity of CNT films with the binder as gauged by Raman spectroscopy.

**Figure 5:**
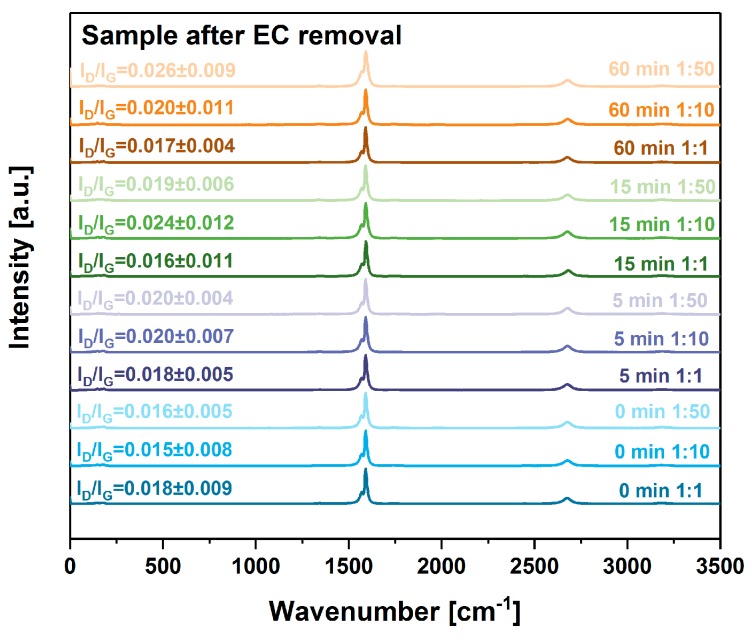
The influence of grinding time and EC content on crystallinity of CNT films after binder removal as gauged by Raman spectroscopy.

Furthermore, we analyzed all the samples by Raman spectroscopy to verify whether the processing had an effect on the crystallinity of the CNTs (Figure 4 and Figure 5) (work of Green and colleagues [34] demonstrated that the destructive impact of sonication could be detected using Raman spectroscopy).

To our delight, neither step (grinding, sonication and removal of EC by thermal annealing) had a negative impact on the degree of structural perfection. Since I_D_/I_G_ ratios indicative of the purity of CNTs, remain virtually unchanged (given the standard deviations), we can postulate that the composition of the CNT material is preserved. Slight variation in I_D_/I_G_ ratios (from 0.01 to 0.02) between the samples can be ascribed to the influence of background noise because there is no clear trend in the data and the D-band is barely visible. As expected, the removal of EC did not affect the purity of the material (Figure 5). Another matter is the length of CNTs, which, under certain circumstances, could be affected without influencing the shape of the spectra. Length variation would have an effect on the ability of material to transport electrical charge. The shorter the CNTs, the higher the scattering rate caused by the increased density of junctions within the network. To study the potential influence of this factor, we measured the electrical and thermal conductivity of the films.

**Figure 6:**
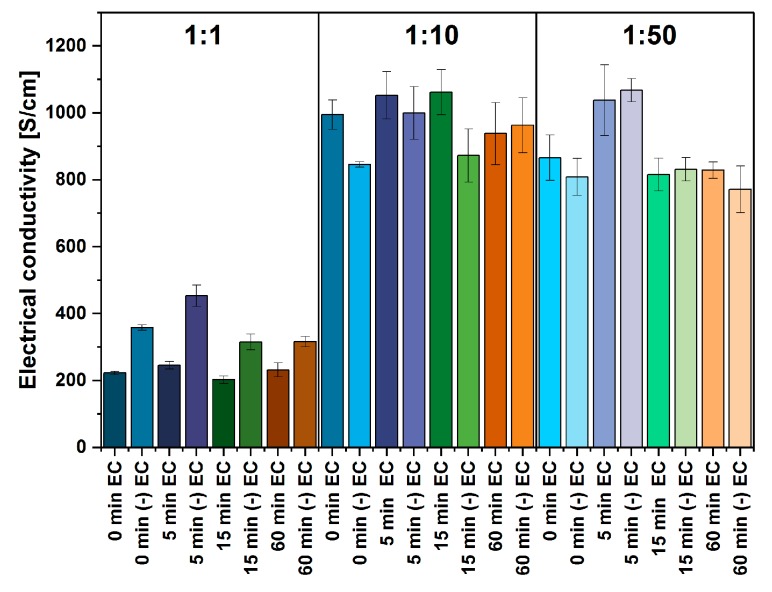
The influence of grinding time and EC content on electrical conductivity (before and after EC removal indicated with (-) prefix).

Regardless of the applied grinding time in the initial stage, SWCNT films with EC in the ratio of 1:1 are characterized by a similar value of electrical conductivity, i.e., ca. 200 S/cm (Figure 6). The amount of insulating polymer binder is very high, which does not enable efficient charge transport through the network. After the removal of EC, these films demonstrate an increase in electrical conductivity in all the cases, which was indicative of improved electrical contact between individual CNTs and their bundles. The biggest changes were recorded for the film obtained from SWCNTs which were first ground for 5 min—its conductivity increased by as much as 85% (from 245 S/cm to 453 S/cm). The film obtained from the input material and ground for 15 min demonstrated an increase by 55% (from 202 S/cm to 315 S/cm), and the one with the longest period of grinding demonstrated a less significant increase—by 35% (from 231 S/cm to 316 S/cm). We observed that grinding may aerate the nanocarbon material, which increases its bulk density. Prolonged processing by this method may not be recommended because air entrapped within the carbon nanotube network deteriorates the electrical conductivity. Even subsequent sonication in liquid medium does not guarantee efficient removal of air between bundles composed of single-walled CNTs.

In summary, the electrical conductivity of 1:1 EC:CNT films is relatively low. Since the films were composed of as much as 50% of EC, upon its removal, the structure of the network is loosely connected. CNTs are separated by a large number of voids (both between individual CNTs and their bundles), which gives a large contact resistance. On the other hand, the application of smaller amounts of EC allows to observe a significant increase in the value of electrical conductivity reaching the level of about 1000 S/cm (for both 1:10 and 1:50 cases). The films obtained from the former EC content (1:10) generally have similar electrical conductivity regardless of the time of grinding. After annealing these samples, we observed a decrease in electrical conductivity. A lack of increase of electrical conductivity is a strong suggestion that percolation pathways in the network have been established at the time of CNT film formation. EC removal can no longer improve the contact between CNTs because its starting content was relatively low and the polymer did not interfere with the electrical transport. On the other hand, flame generated in the course of thermal annealing could contribute to the deterioration of electrical properties of the material. The networks are composed of single-walled CNTs, which have significantly lower thermal stability than double- or multi-walled CNTs [43]. Here, as well, the grinding was beneficial as the electrical conductivity of samples ground for 5 min and 15 min exceeded the conductivity of networks made from unground material. Again, the conductivity of CNT films ground for 60 min was the lowest among all of them. It indicates that such time is excessive and causes unwanted aeration of the material.

Lastly, the films obtained from 1:50 EC content experienced the smallest differences in electrical conductivity before and after annealing. A significantly reduced amount of binder decreased the flame very much, so the influence on annealing on relatively fragile single-walled CNTs was alleviated. The film made of CNTs, subject to grinding for 5 min, demonstrated the highest value of electrical conductivity, i.e., 1067 ± 34 S/cm. It constitutes about four-fold improvement in comparison with the corresponding EC film with 1:1 EC amount relative to CNTs. Prolonged grinding was not beneficial in this case as well.

**Figure 7:**
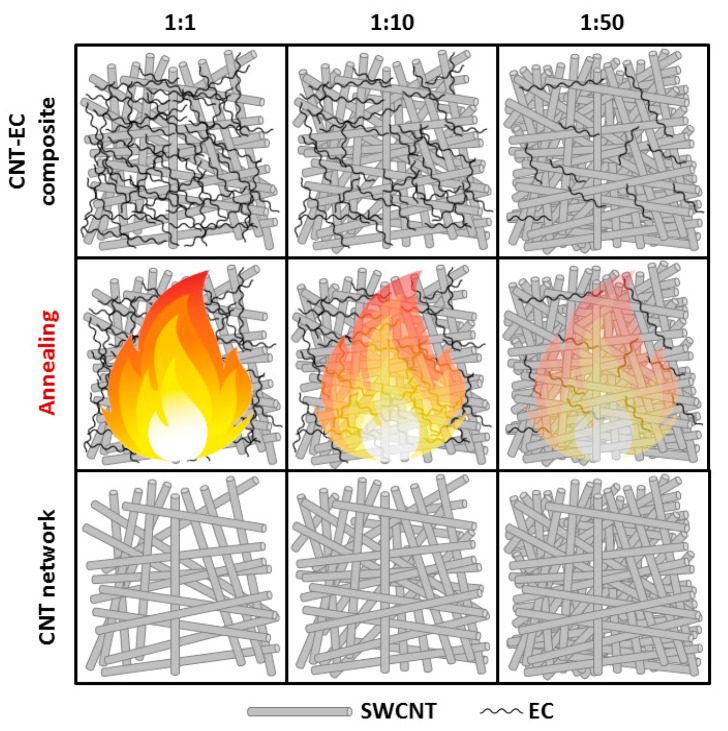
Diagram demonstrating changes in films before and after removal of EC.

These observations may be depicted by differences in structure among the films depending on the applied amount of EC (Figure 7). In the case of the film obtained with 1:1 amount of binder, the value of electrical conductivity of the film was significantly affected by the presence of EC which is an electrical insulator [44]. After the removal, conductivity improved, because it constituted a significant part of the film and blocked paths of conductivity.

The impact of a relatively large flame generated during the annealing process has been overcome by the formation of new contact points between CNTs, and hence the enhancement of electrical conductivity. What regards 1:10 EC content, decreased amount of binder generated a smaller flame. The exposure of single-walled CNTs to flame because of their lower thermal stability results in a slight decrease of electrical conductivity of CNT films upon annealing. Only when the content of EC is further reduced to 1:50, thermal removal of the binder does not give negative consequences. The size of the flame is much smaller and its color is faint (indicative of lower temperature of the process), so the nanocarbon part is not affected. Lastly, the reduction of EC content leads to improvement of packing of CNTs, which has a positive influence on electrical conductivity (the conductive CNT system is well interconnected, the number of junctions is reduced). In light of these results, we can see that 5 min grinding time and 1:50 EC content seems optimal.

**Figure 8:**
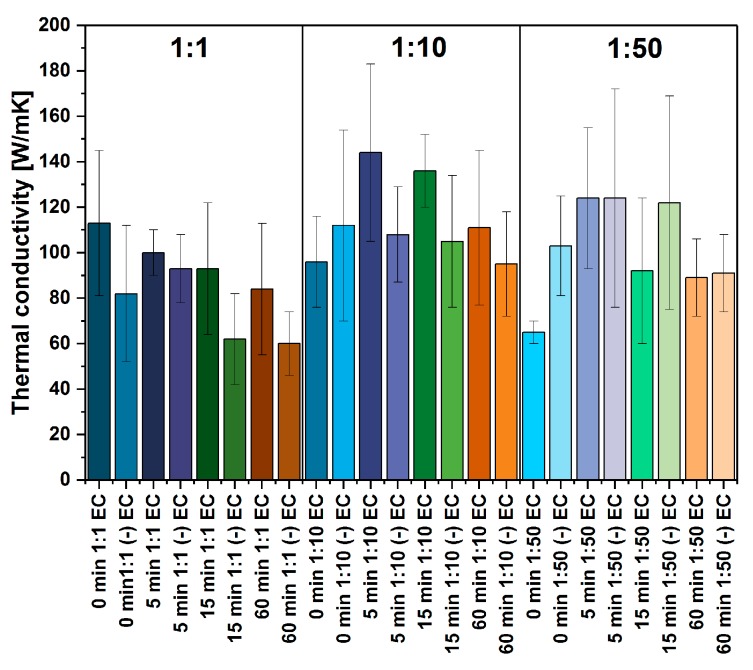
The influence of grinding time and EC content on thermal conductivity (before and after EC removal indicated with (-) prefix).

We have also measured the thermal conductivity of the obtained materials (Figure 8). It is interesting to see that despite drastic changes of electrical conductivity depending on the time of grinding and the amount of EC, thermal conductivity remained generally unaffected in contrast with electrical conductivity (the results are in accordance with similar findings reported by Chen and co-workers [45]). Most samples demonstrate the values of thermal conductivity within the range from 84 W/mK to 113 W/mK. Phonon transport is affected by multiple factors both extrinsic (such as the number of cross-junctions or contact surface between CNTs) as well as intrinsic (e.g., number of CNT walls or presence of defects [5]). On the one hand, the overall thermal conductivity of nanocarbon-based networks can be dominated by extrinsic thermal resistance present within the film [46] (principally when high-quality CNTs are distributed isotropically). On the other hand, intrinsic thermal constraints can be predominant (valid especially for highly aligned films composed of impure CNT material). In our case, the CNT films are isotropic and made from pristine CNTs, which suggests that the former mechanism could be in force. Nevertheless, taking into account the relatively high values of measurement errors (thickness variation, common for CNT ensembles, may be the primary reason), we should be careful with the interpretation of the results. Although the inhomogeneity of the network may not be substantial, steady-state measurement by infrared thermometry, which we used for thermal conductivity determination, is very susceptible to sample size variation. As a consequence, the only notable difference between thermal conductivity values obtained by this method should be at the focal point of thermal analysis. At present, we could conclude that there is no evident influence of the processing parameters or starting composition of CNT films on their thermal conductivity.

## 4. Conclusions

The research conducted by us demonstrated that the correct preparation of parent CNT material has a significant impact on the properties of CNT ensembles formed from it. Simple grinding of the material in a household coffee grinder and optimization of binder content have enabled us to increase the electrical conductivity of the network four times eventually exceeding 1000 S/cm. Such an approach can be very helpful for shortening the time of sonication (commonly used to disperse CNTs) because cavitation can have a negative impact on the microstructure of the material, and hence the properties. In parallel, we observed that the decrease of the binder amount is important because its high content makes the thermal annealing destructive to some extent. The best values of electrical conductivity (1067 ± 34 S/cm) were obtained for the ratio of EC to CNTs of 1:50 and for the duration of grinding in a mechanical grinder of 5 min. The complex nature of thermal transport did not enable us to see a clear influence of the parameters on thermal conductivity of CNT films. 

The search for new methods to enhance the properties of nanocarbon is very important from the strategic point of view. These nanostructures (because of their extraordinary properties) have been envisioned to be next0generation materials for thermal and electrical applications, but their performance has not matched the expectations so far. We have shown that there may be simpler and more convenient alternatives than doping to improve the electrical conductivity of CNT films, which can bring them closer to real-life applications. Since the proposed uncomplicated procedure does not require specialized chemicals or infrastructure, it may be successfully conducted in even the least equipped laboratory. Most importantly, by carrying out such a protocol, we have a guarantee that the electrical conductivity of such a material will not decrease in time, which may happen with a large number of doping agents. Many chemical compounds (in particular halogens, which have been found to strongly dope CNTs) are prone to desorption when exposed to the ambient conditions for a prolonged amount of time or at elevated temperature. Optimization of the CNT network by preprocessing already at the production stage enables us to avoid this problem.

## Supplementary Materials

The following are available online at https://www.mdpi.com/2079-4991/9/8/1113/s1, Figure S1: Coffee grinder (Profi Cook PC-KSW 1021, 200W) used for the study.
