# Supplementary material for: Simple Method to Improve Electrical Conductivity of Films Made from Single-Walled Carbon Nanotubes
Source: Nanomaterials (Basel). 2019 Aug 2;9(8):1113. doi: 10.3390/nano9081113 (PMC6722516; doi:10.3390/nano9081113)
Supplement: Supplementary file 1 [file nanomaterials-09-01113-s001.pdf]

Supplementary information

Simple Method to Improve Electrical Conductivity of Films Made from Single-Walled Carbon Nanotubes

Bogumiła Kumanek [1,*], Tomasz Wasiak [1], Grzegorz Stando [1], Paweł Stando [1], Dariusz Łukowiec [2] and Dawid Janas [1,*]

[1] Department of Organic Chemistry, Bioorganic Chemistry and Biotechnology, Silesian University of Technology, B. Krzywoustego 4, 44-100 Gliwice, Poland

[2] Faculty of Mechanical Engineering, Silesian University of Technology, Konarskiego 18, 44-100 Gliwice, Poland

* Correspondence: Bogumila.Kumanek@polsl.pl, Dawid.Janas@polsl.pl; Tel.: +48-32-237-10-82

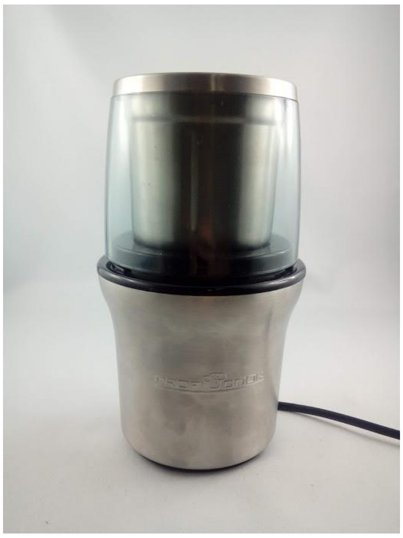

Figure S1. Coffee grinder (Profi Cook PC-KSW 1021, 200W) used for the study.
